# East Asian Young and Older Adult Perceptions of Emotional Faces From an Age- and Sex-Fair East Asian Facial Expression Database

**DOI:** 10.3389/fpsyg.2018.02358

**Published:** 2018-11-29

**Authors:** Yu-Zhen Tu, Dong-Wei Lin, Atsunobu Suzuki, Joshua Oon Soo Goh

**Affiliations:** ^1^Graduate Institute of Brain and Mind Sciences, College of Medicine, National Taiwan University, Taipei, Taiwan; ^2^Department of Psychology, Graduate School of Humanities and Sociology, The University of Tokyo, Tokyo, Japan; ^3^Department of Psychology, College of Science, National Taiwan University, Taipei, Taiwan; ^4^Neurobiological and Cognitive Science Center, National Taiwan University, Taipei, Taiwan; ^5^Center for Artificial Intelligence and Advanced Robotics, National Taiwan University, Taipei, Taiwan

**Keywords:** facial expression database, emotion perception, East Asian, aging, sex

## Abstract

There is increasing interest in clarifying how different face emotion expressions are perceived by people from different cultures, of different ages and sex. However, scant availability of well-controlled emotional face stimuli from non-Western populations limit the evaluation of cultural differences in face emotion perception and how this might be modulated by age and sex differences. We present a database of East Asian face expression stimuli, enacted by young and older, male and female, Taiwanese using the Facial Action Coding System (FACS). Combined with a prior database, this present database consists of 90 identities with happy, sad, angry, fearful, disgusted, surprised and neutral expressions amounting to 628 photographs. Twenty young and 24 older East Asian raters scored the photographs for intensities of multiple-dimensions of emotions and induced affect. Multivariate analyses characterized the dimensionality of perceived emotions and quantified effects of age and sex. We also applied commercial software to extract computer-based metrics of emotions in photographs. Taiwanese raters perceived happy faces as one category, sad, angry, and disgusted expressions as one category, and fearful and surprised expressions as one category. Younger females were more sensitive to face emotions than younger males. Whereas, older males showed reduced face emotion sensitivity, older female sensitivity was similar or accentuated relative to young females. Commercial software dissociated six emotions according to the FACS demonstrating that defining visual features were present. Our findings show that East Asians perceive a different dimensionality of emotions than Western-based definitions in face recognition software, regardless of age and sex. Critically, stimuli with detailed cultural norms are indispensable in interpreting neural and behavioral responses involving human facial expression processing. To this end, we add to the tools, which are available upon request, for conducting such research.

## Introduction

Emotions conveyed in facial expressions are perceived differently by individuals of different cultural backgrounds (Biehl et al., 1997; Jack et al., 2012), age and sex (Hall and Matsumoto, 2004; Isaacowitz et al., 2017), and with different in-group/out-group biases (Lazerus et al., 2016). Neural responses to the same face emotion stimuli also differ across people groups, further supporting the notion that persons from different social or demographic backgrounds have psychologically different experiences of the same emotional signals (St Jacques et al., 2009; Stevens and Hamann, 2012; Hilimire et al., 2014; Gamond et al., 2017). Such neurobehavioral differences in emotional interpretation of facial expressions highlight the need to also consider individual variability in the underlying mental and neural representations of face stimuli that differ by culture, age, or sex (Biehl et al., 1997; Minear and Park, 2004; Kennedy et al., 2009; Ebner et al., 2010).

Many comprehensive emotional stimuli databases are already available that when pooled together consist of young and older, male and female, facial expressions across various ethnic groups (Table 1). Separately, however, these face databases are based on a small number of distinct facial identities, focused mainly on Caucasian face stimuli, focused mainly on young adults, were acquired in non-controlled environmental settings, or validated emotional expressions using ratings by Western-based samples. In addition, expressions depicted in the face stimuli databases predominantly centered their emotional definitions on the Facial Action Coding System (Ekman and Friesen, 1977; Ekman et al., 2002). In the FACS, combinations of facial muscle movements, named Action Units (AUs), comprise different facial emotional expressions. The most common of these expressions include happy, sad, angry, fearful, disgusted, surprised, and neutral expressions that are conceptualized as reflecting basic emotions universal to many cultures (Darwin, 1873; Ekman and Friesen, 1971).

**Table 1:** Non-exhaustive annotated list of face emotion expression stimuli databases.

**No**.	**Reference**	**Database name**	**Ethnicities (N)**	**Age range (N)**	**Sex (N)**	**No. of unique face identities**	**Total no. of photographs**	**Emotion expressions^a^**	**Expression method^b^**	**Validation method^c^**	**Color**
1	Friesen and Ekman (1976)	Pictures of Facial Affect (PoFA)	Caucasian	Not specified	Male (6), Female (8)	14	110	H, Sa, D, F, Su, A, N	FACS	Uni-emotional ratings by Caucasian individuals	Grayscale
2	Mandal (1987)	-	Oriental North Indian (29)	Not specified	Male (15), Female (14)	29	195	H, Sa, D, F, Su, A	Free posing	Uni-emotional ratings by local sample	Not specified
3	Matsumoto and Ekman (1988)	Japanese and Caucasian Facial Expressions of Emotion (JACFEE)	Japanese (28), Caucasian (28)	Not specified	Male (28), Female (28)	56	56	H, Sa, D, F, Su, A, N	FACS	Uni-emotional ratings across several countries	Color
4	Mazurski and Bond (1993)	-	Not specified	8–12 years (6), 18–40 years (10)	Male (8), Female (8)	16	175	H, Sa, D, F, Su, A, N	Free posing	Uni-emotional ratings by local student sample	Color
5	Lundqvist et al. (1998)	Karolinska Directed Emotional Faces (KDEF)	Caucasian	20–30 years (70)	Male (35), Female (35)	70	4,900	H, Sa, D, F, Su, A, N	Free posing	-	Color
6	Lyons et al. (1998)	-	Japanese (10)	Not specified	Female (10)	10	219	H, Sa, D, F, Su, A, N	Free posing	Multi-emotional ratings by local female student sample	Grayscale
7	Wang and Markham (1999)	-	Chinese (17)	20–28 years (17)	Male (10), Female (7)	17	75	H, Sa, D, F, Su, A	Free posing	Uni-emotional ratings by local student sample	Grayscale
8	Beaupré et al. (2000)	Montreal Set of Facial Displays of Emotion (MSFDE)	French Canadian (8), Chinese (8), sub-Saharan African (8)	Not specified	Male (12), Female (12)	24	144	H, Sa, D, F, A, N	FACS	Multi-emotional ratings by local sample	Grayscale
9	Gur et al. (2002)	-	Caucasian (91), African American (23), Asian (6), Hispanic (10)	10–85 years (139)	Male (70), Female (69)	139	5,560	H, Sa, D, F, A, N	English method; Evoked	Uni-emotional ratings by local sample	Color
10	Minear and Park (2004)	-	Caucasian (435), African American (89), Others (52)	18–29 years (219), 30–49 years (76), 50–69 years (123), 70–93 years (158)	Male (219), Female (357)	576	1,142	Smile, N, profile	Free posing	Not specified	Color
11	Chen and Yen (2007)	Taiwanese Facial Expression Image Database (TFEID)	Chinese (20)	Not specified	Male (20), Female (20)	20	7,200	H, Sa, D, F, Su, A, N	Not specified	Not specified	Color
12	Gao et al. (2008)	CAS-PEAL	Chinese (1,040)	18–74 years (1,040)	Male (595), Female (445)	1,040	99,594	N, Smile, Frown, Su, Close eyes, Open mouth	Free posing	Database for face recognition without known emotion-related validation	Color
13	Tottenham et al. (2009)	NimStim	Caucasian (25), Latin American (2), African American (10), Asian (6)	21–30 years (43)	Male (25), Female (18)	43	646	H, Sa, D, F, Su, A, Calm, N	Controlled posing	Uni-emotional ratings by local sample	Color
14	Tracy et al. (2009)	University of California, Davis, Set of Emotion Expressions (UCDSEE)	Caucasian (2), West African (2)	Not specified	Male (2), Female (2)	4	46	H, Sa, D, F, Su, A, N, Shame, Embarrassment, Pride	FACS; additional guidelines for self-conscious emotions; upper half body postures	Uni-emotional ratings by local student sample	Color
15	Ebner et al. (2010)	FACES	Caucasian (179)	19–31 years (58), 39–55 years (56), 69–80 (57)	Male (86), Female (85)	171	2,052	H, Sa, D, F, A, N	FACS	Uni-emotional ratings by local sample Multi-emotional ratings by local sample in Riediger et al. (2011)	Color
16	Langner et al. (2010)	Radbound Faces Database (RFD)	Caucasian (49)	Adult (39), Children (10)	Male (24), Female (25)	49	5,880	H, Sa, D, F, Su, A, N, Contempt	FACS	Uni-emotional ratings by local sample	Color
17	Zhang et al. (2010)	PolyU Near Infrared Face Database (PolyU-NIRFD)	Chinese (350)	Not specified	Not specified	350	35,000	Not specified	Free posing	Database for face recognition without known emotion-related validation	Grayscale
18	Flanagan (2011)	Face Recognition Technology (FERET)	Not specified	Not specified	Not specified	1,199	14,126	Not specified	Not specified	Database for face recognition without known emotion-related validation	Color
19	van der Schalk et al. (2011)	Amsterdam Dynamic Facial Expression Set (ADFES)	Caucasian (12), Mediterranean (10)	18–25 years	Male (12), Female (10)	22	648 (videos)	H, Sa, D, F, Su, A, N, Contempt, Embarrassment, Pride	FACS	Uni-emotional ratings by local sample	Grayscale
20	Kaulard et al. (2012)	Max Planck Institute (MPI) Facial Expression Database	Caucasian (19)	20–30 years	Male (9), Female (10)	19	19,152	56 different expressions including H, Sa, D, F, A, N	Controlled posing	Uni-emotional ratings by local sample	Color
21	Chen et al. (2013)	-	Chinese (29)	18–53 years (28), 67 years (1)	Male (15), Female (14)	29	2,273	H, Sa, D, F, Su, A, N	FACS	Multi-emotional ratings by local sample	Color
22	Olszanowski et al. (2015)	Warsaw Set of Emotional Facial Expression Pictures (WSEFEP)	Caucasian (30)	20–30 years	Male (14), Female (16)	30	210	H, Sa, D, F, Su, A, N	Controlled posing using Stanislavski acting method; selected using FACS	Multi-emotional ratings by local sample	Color

a *H, Happy; Sa, Sad; D, Disgust; F, Fearful; Su, Surprise; A, Anger; N, Neutral*.

b *FACS, Facial Action Coding System*.

c *Uni-emotional rating approaches require raters to select the single best emotion to categorize face stimuli; multi-emotional rating approaches require raters to indicate the extent to which a given face stimulus portrays various different emotion categories*.

In recent studies, however, whereas Western participants categorized the above six basic emotions (plus neutral expression) consistently with regard to FACS generated face expression stimuli, emotion categorizations in East Asians were notably distributed across the different FACS face expression sets (Jack et al., 2009, 2012, 2016; see Gendron et al., 2014 for differences in Hinda participants as well). Interestingly, eye-tracking work has shown that Western and East Asian participants adopt different viewing strategies when processing face stimuli as well (Blais et al., 2008; Caldara et al., 2010; Gobel et al., 2017). Moreover, functional neuroimaging studies have reported that even in basic passive viewing of faces, engagement of the fusiform face area is more bilateral in Westerners but more right-lateralized in East Asians (Goh et al., 2010a). These findings contend that the heterogeneities in emotional distinctions of facial expressions at least between East Asian and Western groups are not trivial and there are heuristic differences in basic face feature processing. As such, broader definitions of emotional categories and associated facial expression experimental materials are necessary to accommodate the rich variability of neurobehavioral emotion and perceptual processes across different social groups (Henrich et al., 2010).

Here, we considered that a possible account for differences in East Asian and Western categorization of face emotion expressions is the differential emphasis on collectivism and individualism, respectively (Nisbett, 2003). A value for collectivism might bias East Asians to attend more holistically to distinctive facial features (Goh et al., 2010a), which in turn results in more distributed judgments of face emotions that have more overlapping features. By contrast, a value for individualism in Westerners might be associated with a more analytic attention to facial features that emphasizes distinctive features and results in more discretized perceptions of different face emotions. If so, we hypothesized that the previously shown distributed face emotion judgments in East Asian individuals (Jack et al., 2012) should be organized such that face expressions with shared features (AUs in the case of FACS expressions) would tend to be categorized together. As such, multi-dimensional judgments of the extent to which face expressions contain different degrees of different emotions in East Asian raters should cluster in separate emotion categories to the extent that face expression features in the stimuli do not overlap. The different AUs associated with the above basic face emotion expressions are listed in Supplementary Table 1, based on Ekman et al. (2002). In these expression definitions, happiness is most distinctive, having a unique AU 12 and only has one AU in common with sadness. By contrast, fearful and surprised faces share five AUs with AU 27 unique to them both. Angry and disgusted faces share four AUs with AU 10 unique to them both. Sad and fearful faces show some overlap with each other and also with angry and disgusted faces. Thus, we expected that East Asians would identify happy faces as a distinct face emotion category. However, East Asian face emotion perception might group fear and surprise as one category, and anger and disgust as one category. For sadness and fear, East Asians might consider these expressions as distributed between the above two categories or as a fourth category with partial overlap with either categories. Overall, multi-dimensional emotion judgments should reflect three or four, rather than six, categories of emotion perceived by East Asians when rating face expressions based on the FACS approach (Jack et al., 2016).

Apart from culture-related differences, age and sex differences within a culture group also modulate face expression perception. Compared to younger adults, older adults have reduced neural fidelity in processing facial features (Goh et al., 2010b) and show lower performances in face emotion identification and recognition (Sullivan and Ruffman, 2004; Ruffman et al., 2008; Ebner and Johnson, 2009; Ebner et al., 2011; Suzuki and Akiyama, 2013; Sullivan et al., 2017). Moreover, there is a positivity bias in older adults so that age differences are accentuated for negative stimuli (Mather and Carstensen, 2005; Riediger et al., 2011; Di Domenico et al., 2015; Franklin and Zebrowitz, 2016). In addition, females have greater sensitivity than males to facial expressions that is also with accentuated effects for negative stimuli (Rotter and Rotter, 1988; McClure, 2000; Thayer and Johnsen, 2000; Hall and Matsumoto, 2004; Montagne et al., 2005; Suzuki et al., 2006; Williams et al., 2009; Sullivan et al., 2017) and accompanied by differences in neural activity (Stevens and Hamann, 2012). Moreover, sex differences in face emotion processing have been shown to extend to old age (Demenescu et al., 2014; Sullivan et al., 2017). Also, the age-related positivity bias effect differs between Westerners and East Asians in face emotion processing (Fung et al., 2008; Ko et al., 2011) as well as for non-facial emotional stimuli (Kwon et al., 2009; You et al., 2009; Grossmann et al., 2014; Zhang and Ho, 2015).

At present, however, data are still relatively scant on whether the above age or sex differences in face emotion perception, examined mostly in Western-based samples, are replicated in East Asian samples. Thus, we were interested in evaluating whether an East Asian categorization pattern of face emotion expressions as described above would be consistent between young and older, male and female adults. With respect to age, prior studies have found that culture-related differences in cognitive processes involving visual scenes and semantic categorization are generally maintained or even accentuated in older adults (Chua et al., 2006; Gutchess et al., 2006; Yoon et al., 2006; Goh et al., 2007; Yang et al., 2013). Thus, we expected that the pattern of multi-dimension face emotion categorization as seen in young adult East Asians would also generally not diminish in older adults, reflecting the robustness of the East Asian style of face emotion perception with age. Nevertheless, it is also possible that older adult East Asians might show positivity bias that differentially affects ratings for happy relative to the other face emotions. In addition, because greater age is associated with reduced sensitivity to overall facial emotions as reviewed above, there should be generally lower emotional intensity ratings in older relative to younger adults (see also Suzuki et al., 2005; Orgeta and Phillips, 2008). With respect to sex, because of the prior evidence above as well, we expected greater sensitivity to facial expressions in females than males that is associated with overall higher emotion intensity ratings. However, again, we did not expect sex differences to diminish the multi-dimension face emotion categorization pattern seen in this East Asian sample as a whole. Of additional interest, we considered the potential influence of interpersonal collectivism in East Asian perceptual processing (Goh and Park, 2009; Grossmann et al., 2014) on in-group/out-group biases to do with rater and face stimuli age and sex. Specifically, in studies with Western-based samples, participants perceived in-group faces as displaying more positive affect than out-group faces (Lazerus et al., 2016), reflecting the emphasis of dissociating self from others. In our Taiwanese sample, however, we expected that a more collectivistic emphasis would minimize our ability to detect differences in participant face emotion judgments between same- and other- age and sex stimuli.

In this study, we present and evaluate a set of East Asian face emotion stimuli based on expressions enacted by Taiwanese young and older adult males and females. We used the FACS criteria (Ekman and Friesen, 1977; Ekman et al., 2002) under systematic controlled settings for photography to generate our emotional face stimuli. Thus, our stimuli are comparable to extant face databases using similar methodology and can in theory be integrated as needed in experimental applications. As such, our validation approach combined face photographs acquired in this present study as well as photographs from Chen et al. (2013) acquired using similar methods. In addition, our young and older East Asian faces were based on natural photographs involving real expressions that better capture true variation in human expressions compared to computer generated stimuli (Jack et al., 2012, 2016). To evaluate the above expectations, during validation rating, we assessed how local individuals perceived the six basic emotions in our face stimuli using multi-dimensional emotional ratings for each face stimuli rather than the uni-dimensional, best emotion category approach. In addition, we evaluated how each emotional face induced positive or negative affective experience using self-report in raters. This afforded a means to assess the effectiveness of our stimuli in inducing affective reactions as well as to evaluate distinctions between face emotion recognition performance and face emotion subjective reactions. In addition, such norms on subjective reactions to the stimuli might be of use in some future studies. Critically, to validate that visual features associated with the different basic emotion categories were present in our Taiwanese face stimuli, we applied a commercial face emotion recognition software that was developed using the FACS criteria (Face Reader, Noldus Information Technology, The Netherlands; Langner et al., 2010) and compared the algorithm's performance against our human raters. Overall, our study provides comprehensive evaluations of multivariate emotional reaction profiles to Taiwanese face stimuli in Taiwanese young and older, male and female participants that are based on but not restricted to FACS criteria. Face stimuli along with the rating profile norms are available upon request.

## Methods

### Facial expression stimuli

#### Participants

Taiwanese facial emotional expression stimuli in this study comprised a combination of photographs from an existing database (Chen et al., 2013) and photographs acquired in this present study. Stimuli from the Chen et al. (2013) database included a total of 1,232 frontal view face emotion expression photographs of 29 unique Taiwanese actors (15 males, 14 females) expressing six basic emotions (happiness, sadness, disgust, fear, surprise, and anger) and neutral expression based on the FACS approach, acting method, and free posing. We note that although the age range of face actors in the Chen et al. (2013) database spanned young to older adults, there was a lower representation of older adults (see Table 1, no. 21).

To balance the representation of young and older adults faces and to increase the number of unique identities, we recruited 20 young adults [mean age (SD): 23.4 (2.3) years; age range: 20–28 years; 10/10 males/females] and 41 older adults [mean age (SD): 67.6 (6.7) years; age range: 58–86 years; 20/21 males/females] for photo collection. All participants were Taiwanese living around the Taipei area at the time of photography, provided written informed consent for the use of their face photographs for academic purposes, and were remunerated for their time. The Research Ethics Committee at the National Taiwan University Hospital approved this photo collection study.

#### Photo collection procedure

Upon arrival at the laboratory, participants were instructed on the face emotion expression and photography procedure. This included brief introduction and training with photographic and live examples by the experimenter on AUs and emotion predictions as described in the FACS (Ekman et al., 2002). Training material was translated and conducted in Mandarin for our Taiwanese participants. After training, participants removed eyeglasses and fixed their hair to expose the forehead and eyebrows using hairpins as needed. Participants then sat on a chair against a white background facing a digital mirrorless interchangeable lens camera (Nikon 1 J1) placed one meter away that was fixed on a tripod. Camera height was adjusted so that participants' faces occupied the central area of the photographs. Lighting in the photography area was applied using standard white fluorescent lamps and was the same across all participants. Color photographs were then taken as each participant made facial emotion expressions first using the FACS AU method and then using free posing. Thus, participants generally contributed more than one facial photograph depicting each basic emotion that included happy, sad, disgusted, fearful, angry, surprised, and neutral expressions. Supplementary Table 1 shows AUs from the FACS defining each of the basic emotions (sans neutral) as well their unique and common features. As mentioned above, in Supplementary Table 1, the FACS definition of happiness is most distinctive with 50% of its AUs in common with sadness. All other emotions had higher proportions of shared AUs across emotions in general. In particular, anger and disgust share four AUs, surprise and fear share five, and sadness shares AUs relatively distributed across the other emotions. Moreover, fear has no unique AUs. Readers interested in further details regarding each AU are referred to Ekman et al. (2002). The total initial pool of photographs taken was 2,477, each with 2,592 × 3,872 pixels resolution.

#### Face stimuli preprocessing

Face photographs from our own study and from Chen et al. (2013) were combined yielding a total of 3,709 photographs. These photographs were converted to grayscale using Matlab R2012b (MathWorks, Natick, MA, United States) to reduce the effects of different skin tones. A black foreground frame with an oval cutout was then applied onto the grayscale photographs using Adope Photoshop® CC 2014 (Adobe Systems Incorporated, San Jose, CA, United States) to exclude the hair, ears, and neck. Finally, contrast and brightness of the framed grayscale photographs were normalized using a Matlab script (https://www.mccauslandcenter.sc.edu/crnl/tools/bmp__contrast).

A subset of the preprocessed photographs taken with FACS AU method was selected for further validation rating. Photographs in which face movements during emotion expression were overly exaggerated and compromised the photo were excluded. Also, the experimenters (YZT and DWL) who trained the participants on the FACS as described in Ekman et al. (2002) selected those photos in which the AUs expressed by participants clearly met the FACS criteria based on their subjective judgment. We note that while the experimenters were not officially trained in the FACS, their judgement was based on experience with descriptions stated in the FACS, and subsequent application of the stimuli using commercial software (see below) was able to cluster the photographs according to the assigned emotion expression categories, validating the face emotions present in the stimuli. The resulting selected age- and sex-fair Taiwanese facial expression database consisted of 628 photos, which includes 90 unique identities each with seven assigned basic face emotion expression categories, excluding two ill-posed faces. Note also that one identity had two different angry expressions and no sad expression. Among the 90 identities, 48 are young [mean age (SD): 28.5 (9.9) years; age range: 18–51 years; 23/25 males/females] and 42 are older [mean age (SD): 67.5 (6.6) years; age range: 58–86 years; 21/21 males/females] adults. These photographs were then submitted to the rating experiment for validation and to obtain norms of the emotional expressions perceived by local Taiwanese.

### Facial expression stimuli human validation

#### Participants

Twenty young [mean age (SD): 24.0 (2.8) years; age range: 20–30 years; 10/10 males/females] and 24 older [mean age (SD): 70.2 (6.7) years; age range: 59–86 years; 8/16 males/females] adults were recruited from the local community in Taipei, Taiwan for rating validation of the face photograph stimuli. All participants scored 26 or above in the Mini-Mental State Examination (Folstein et al., 1975; Guo et al., 1988) as part of the screening criteria. All the participants were remunerated for their time and provided written informed consent for this study, which was approved by the Research Ethics Committee at the National Taiwan University Hospital.

#### Rating experiment procedure

**Figure 1:**
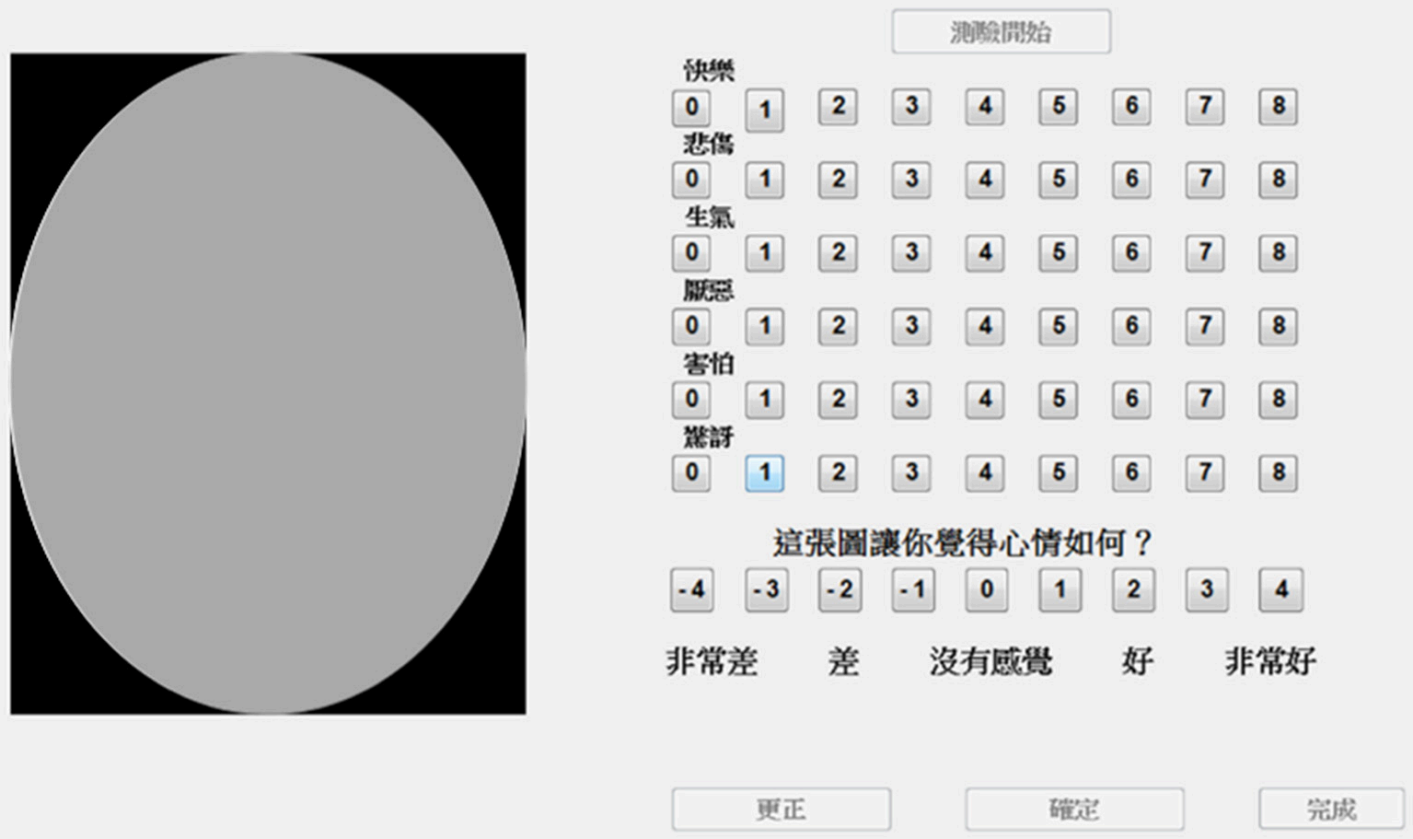
The graphic user interface used for the rating experiment. A face photo was depicted on the left (in place of the gray oval; face not shown due to usage permissions issues), and a rating scale on the right. Participants were required to rate each face photo for all six dimensions of emotion. Ratings of 0 to 8 indicated the lowest to highest intensity of the dimension of emotion, respectively. In addition, participants also rated the subjective valence they experienced due to viewing the photo. Rating of −4 to 4 indicated very bad to very good affect experienced, respectively, with 0 indicating neutral affect. (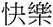: Happiness, 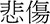: Sadness, 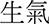: Anger, 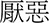: Disgust, 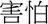: Fear, 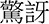: Surprise; 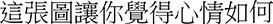
?: How does this picture make you feel?; 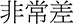: Very bad, 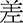: Bad, 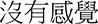: Nothing, 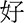: Good, 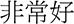: Very good).

The graphic user interface for the rating experiment was created using Psychtoolbox ver. 3.0.14 (http://psychtoolbox.org/) running on Matlab and presented using a desktop computer running Windows 7. In each trial, participants saw the processed face stimuli on the left of the computer screen along with the rating scales on the right (Figure 1). Rating scales were provided for each of the six basic emotion dimensions (happiness, sadness, anger, disgust, fear, and surprise). Face emotion rating scales ranged from 0 to 8 indicating extremely low to extremely high intensity of the specific emotion, respectively. A subjective affect rating scale was also included below the face emotion rating scales that ranged from −4 to 4 indicating very bad to very good feeling experienced due to viewing the face photograph (0 being neutral affect).

The 628 face photographs for validation rating were distributed into two separate lists of 314 faces, each consisting of all 90 identities. List assignment was counterbalanced across participants, separately for young and older adults. Participants were allowed to take breaks as needed around the laboratory premises to minimize effects of fatigue on ratings. Four participants did not finish the rating task including three older females who rated 238, 95, 43 photographs, respectively, and one old male who rated 313 photographs. These incomplete data were still included in subsequent analyses and norms accordingly. Participants were instructed to rate every facet of emotions that they judged was seen in the face photographs by indicating the intensity score of the six basic emotions using mouse button presses accordingly. Participants were also instructed to rate their own feeling valence after watching the face stimulus. Each trial had unlimited duration and the rating experiment was conducted in a dedicated testing room.

#### Data analysis

Data visualization and analyses were done using R ver. 3.3.3 (R Core Team, 2017) with additional package libraries including ggplot2, plot3D, animation, MCMCglmm, gridExtra, and magrittr. Mean emotion ratings for each assigned face emotion expression category were plotted as radial graphs to visualize the multi-emotional profiles as perceived by our Taiwanese participants. To evaluate the simultaneous dimensionality of face emotion categorizations in our Taiwanese participants, we applied multidimensional scaling (MDS) on the multi-emotional ratings provided by the raters. Specifically, we computed Manhattan distances between all face stimuli using all the associated 6-dimensional emotion profiles indicated by all raters. Manhattan distances were used as the measure of statistical distances here because the integer ratings used by raters were discrete rather than continuous. We then used MDS to project these distance relationships onto a 3-dimensional space to visualize the cluster distribution and membership of the different face emotion stimuli. Hierarchical clustering was further applied to more quantitatively corroborate the observed cluster distribution in the MDS analysis. Specifically, we applied a cut off of seven clusters in the hierarchical cluster grouping and assessed the percentages of face stimuli in each face emotion category that were also in each cluster. Face stimuli that were clustered together indicated that they were rated similarly across participants. Moreover, to the extent that face stimuli membership with the seven assigned emotion categories correspond with the resulting clusters indicates that Taiwanese perceive the basic emotions as by defined using the FACS approach. Both MDS and hierarchical clustering analyses were applied to the entire rating data. MDS was also applied separately for young and older adults, males and females, to also visualize age and sex differences in face emotion perception.

To formally test rating differences due to assigned face emotion categories and the contributions of age and sex differences, we used a Markov Chain Monte Carlo (MCMC) approach to evaluate linear mixed models of the multivariate rating behavior as the dependent variable and categorical emotion stimuli as an independent variable (Hadfield, 2010). MCMC models used 13,000 iterations including 3,000 burn-in ones and a thinning interval of 10. Gaussian distribution of the data and residual covariance independence were assumed. Prior specifications of variance structure were diagonal matrices reflecting independence between dependent variables.

For emotion perception of faces, the multivariate MCMC mixed model included the rated intensities of the six emotion dimensions as the dependent variables. Fixed effects included the assigned face emotion category (Happiness, Sadness, Disgust, Fear, Anger, Surprise, or Neutral), subject age (young or older rater), subject sex (male or female rater), and the full interactions between them. In addition, face age (young or older poser), face sex (male or female poser), and their respective interactions with subject age and subject sex under each assigned emotion category were considered to control for in-group/out-group effects of age and sex accordingly. The intercept was inhibited for parsimony. Different faces and participants were set as random effects (Supplementary Methods, Equation 1). For induced affect, the MCMC model included the rated negative to positive affective scores as the dependent variable. Fixed effects were the six rated emotion dimension intensities, subject age, subject sex, and the interactions between each dimension and age or sex. Random effects were the same as in the previous model (Supplementary Methods, Equation 2).

### Facial expression stimuli validation by computer software model

The 628 face emotion stimuli were passed through Face Reader software (Noldus Information Technology, Netherlands), which generated multi-emotional intensity ratings based on algorithms that modeled the FACS criteria. The East Asian module was used to optimize the emotion recognition algorithm for processing the Taiwanese face features in our study. Fourteen out of the 628 stimuli (one neutral, one happy, one sad, two angry, three disgusted, one fearful, and five surprised faces) could not be processed by Face Reader. This was because lighting, contrast, and composition limitations in the framed stimuli limited Face Reader's ability to accurately parse the boundaries and physical features of the faces. Generated emotion ratings based on the rest of 614 stimuli for each assigned face emotion category were plotted as radial graphs to visualize the multi-emotional profiles as detected by the software model. Similar to human ratings, we applied MDS and hierarchical clustering on the multi-emotional ratings provided by Face Reader. Specifically, MDS projected the Euclidean distance relationships of the ratings onto a 3-dimensional space to visualize the distribution of clusters of the different face emotion stimuli as detected by Face Reader. Euclidean distance was used as the statistical distance index here as this was more appropriate for the float ratings on a continuous scale from 0 to 1 provided by Face Reader.

## Results

### Taiwanese dissociated fewer than six basic emotions in face expression stimuli

**Figure 2:**
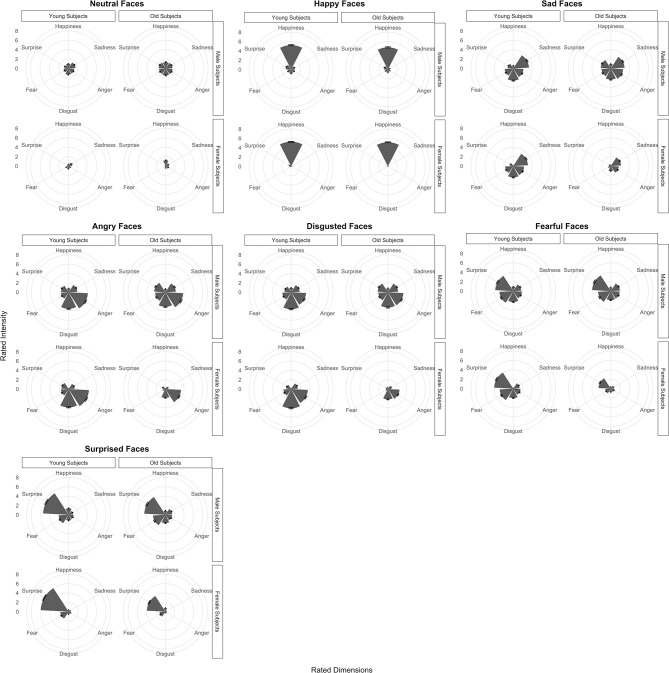
Radar charts showing multidimensional profiles of mean Taiwanese participant ratings across six emotions (happiness, sadness, anger, disgust, fear, and surprise) for happy, sad, angry, disgusted, fearful, surprised, and neutral face expression stimuli categories. Ratings are separated into young and old, male and female groups. Error bars denote standard errors.

Mean multi-emotional rating profiles for each assigned face emotion category are illustrated as separate radar graphs for young and old, male and female participants in Figure 2 and formally tested using multivariate MCMC mixed-model regression (Supplementary Table 2). As expected, ratings of emotional intensities for the neutral face category were uniformly low across all emotion dimensions with mean ratings ranging from 0.9 to 1.6 (95% C.I.: 0.2–1.6 and 0.9–2.3, respectively; Supplementary Table 2, rows 1–6). Emotional profiles for the happy face category were clearly selective for the emotion with mean rating of 5.4 for the happiness dimension and < 1.0 for all other dimensions except surprise (Supplementary Table 2, rows 7–12). The emotional profile for sad faces loaded most on the sadness dimension (3.7), but also loaded somewhat on anger, disgust and fear (1.9, 2.6, and 1.9, respectively) and was least distinctive next to neutral faces (Supplementary Table 2, rows 13–18). By contrast, emotional profiles for angry and disgusted faces appeared to load similarly on these two emotional dimensions (> 3.0) but also included weightings of sadness, fear, and surprise (>1.0, except for happiness; Supplementary Table 2, rows 19–30). Also, emotional profiles for fearful and surprised faces both loaded mainly on surprise (>4.0) but included weightings of sadness, anger, disgust, and fear as well (>1.0, except happiness for fearful faces and sadness for surprised faces; Supplementary Table 2, rows 31–42). We note that the above statistical tests of rating differences applied to young males as the baseline, although the relative multi-emotional profiles in young females, and older males and females were generally similar. We also note that the ratings quite closely reflected the unique and common AUs associated with each assigned face emotion category based on the FACS (Supplementary Table 1). Differences in ratings due to age and sex relative to these young male responses are evaluated in the next section.

**Figure 3:**
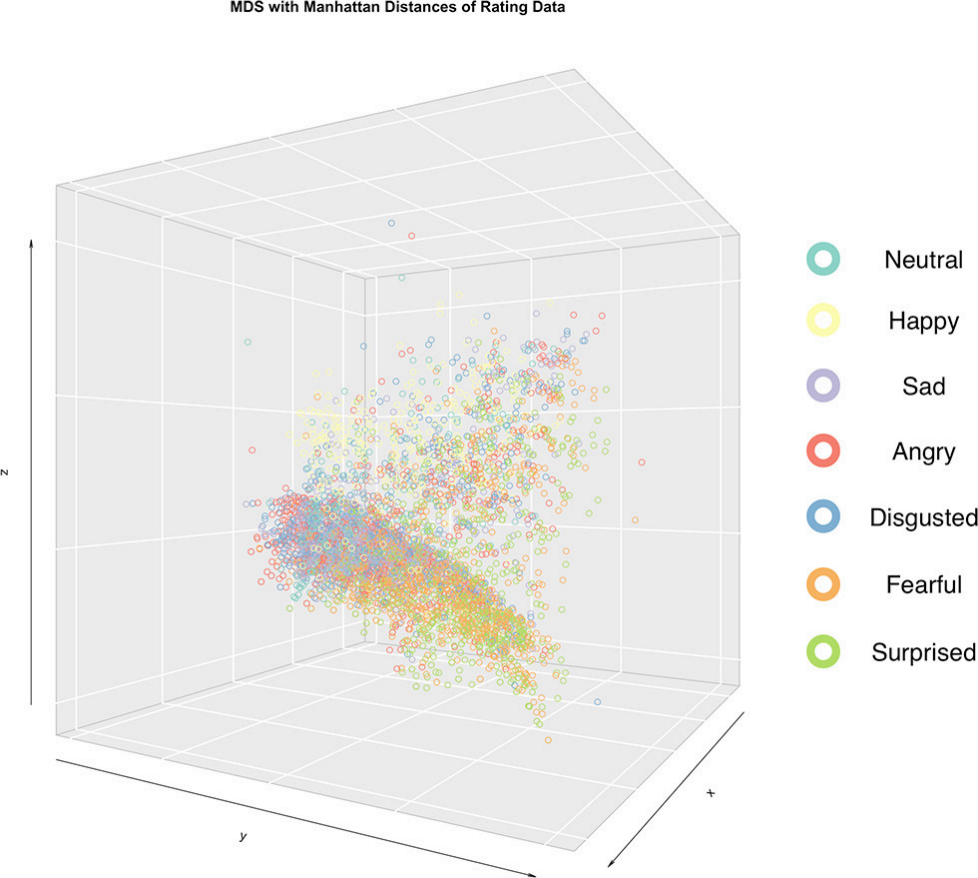
Emotion rating profiles (six emotion dimensions) for each face stimuli from each rater were projected in a 3-D space based on multi-dimensional scaling (MDS) analysis. Face stimuli positions in the 3-D space were determined using Manhattan distances (see Methods). Face stimuli position markers were color-coded according to the assigned emotion category from the FACS approach.

To corroborate the above observations, statistical distances between face stimuli were computed from the multi-emotional profile ratings and projected into 3-dimensional space using MDS for all participants jointly (Figure 3 and Supplementary Movie 1). Face stimuli in the 3-dimensional space were color-coded for their assigned emotion category. As can be seen, three general clusters were dissociated with the following groupings of (1) happy, (2) sad, angry, and disgusted, (3) fearful and surprised faces. Moreover, this clustering pattern was the same across age and sex groups (Supplementary Movies 2–5).

**Figure 4:**
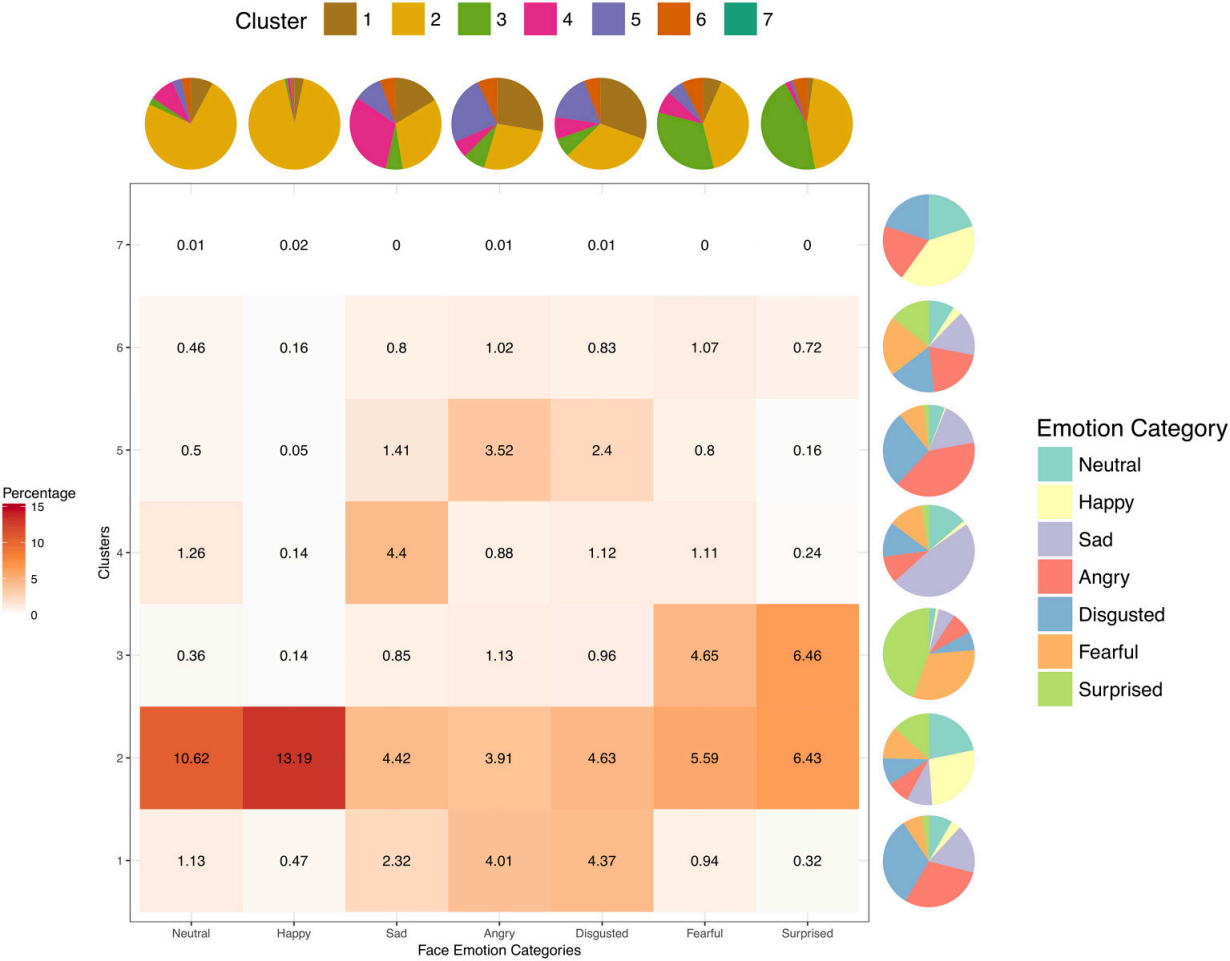
The heat map depicts percentages of all face stimuli in each of the seven face categories for each cluster obtained from hierarchical clustering analysis (cut off at seven clusters) of Taiwanese participants' multi-dimensional ratings. The pie charts represent the marginalized proportions of composition within each cluster (next to each row of the heat map) or face emotion category (above each column of the heat map).

Figure 4 shows the percentage cluster and face emotion category memberships from hierarchical clustering of participants' multi-dimensional emotion ratings cut off at seven clusters (see Supplementary Figure 1 for clustering dendrogram), and also the respective proportion marginalization of each cluster and face emotion category. As can be seen in Figure 4, cluster membership did not dissociate according to face emotion category. Rather, cluster 2 represented the largest proportion of faces with highest representations for neutral and happy faces, albeit substantial representations were also present from the other categories. Cluster 1 captured face expressions from sad, angry, and disgusted faces, whereas cluster 3 captured fearful and surprised faces. Face stimuli memberships in clusters 4, 5, and 6 reflected the ambiguity between sad, angry, disgusted, and fearful faces. Thus, as with the radar graphs and MDS, hierarchical analysis again dissociated only (1) happy faces (cluster 2), (2) sad, angry, and disgusted faces (cluster 1, 2, 4, and 5), and (3) fearful and surprised faces (cluster 2 and 3) in Taiwanese face emotion ratings. Overall, instead of six distinct face emotion categories, Taiwanese participants considered Taiwanese sad, angry, and disgusted expressions as alike, fearful and surprised expressions as alike, and happy faces as distinct from these other two sets of face expressions.

### Age and sex differences in face emotion perception

While there were no significant age and sex differences in emotion dimension ratings when considering all face emotion categories together, there were several specific age sex interactive effects across different face emotion categories. Specifically, compared to young males, older males gave significantly lower emotion dimension ratings for the target emotion dimension of each face emotion category (Supplementary Table 2, rows 55, 62, 69, 76, 83, and 90), whereas young females gave significantly higher emotion ratings (Supplementary Table 2, rows 91, 98, 105, 112, 119, and 126). Critically, compared to older males and young females, older females gave significantly lower ratings of sadness, anger, disgust, fear, and surprise dimensions but no significant difference for the happiness dimension ratings (Supplementary Table 2, rows 133, 140, 147, 154, 161, and 168). These findings suggest that Taiwanese young females generally perceive higher intensities of emotions in face expressions than young males do. However, age reduces the perception of emotional intensity especially for Taiwanese older females and particularly for negative affect expressions.

### Effects of age and sex of face expressions and in-group/out-group biases

Young and older adults perceived older faces as having less distinctive emotional intensity. Specifically, both young and older adults gave higher ratings of happiness for older than young neutral faces (Supplementary Table 2, rows 169 and 211). In addition, both young and older adults also gave lower target dimension ratings of older than younger happy, sad, and angry faces, with the differences also seen for fearful faces in young adults (Supplementary Table 2, rows 175, 182, 189, 203, 217, 224, and 231). Thus, our findings suggest that Taiwanese older face expressions contain less intensity of emotional information. Moreover, Taiwanese young and older adults similarly perceived these differences suggesting minimal age-related in-group/out-group biases on face emotion perception.

We also found minimal sex-related in-group/out-group biases, with males rating female fearful faces as expressing less surprise only (Supplementary Table 2, row 288). There were no other significant differences in other-sex target dimension ratings of face expression categories when males rated female faces, or when females rated male faces. Interestingly, we noted that both males and females rated female surprised faces as expressing less fearfulness than male surprised faces (Supplementary Table 2, rows 293 and 335).

### Age and sex modulated the influence of face expressions on subjective affect

**Figure 5:**
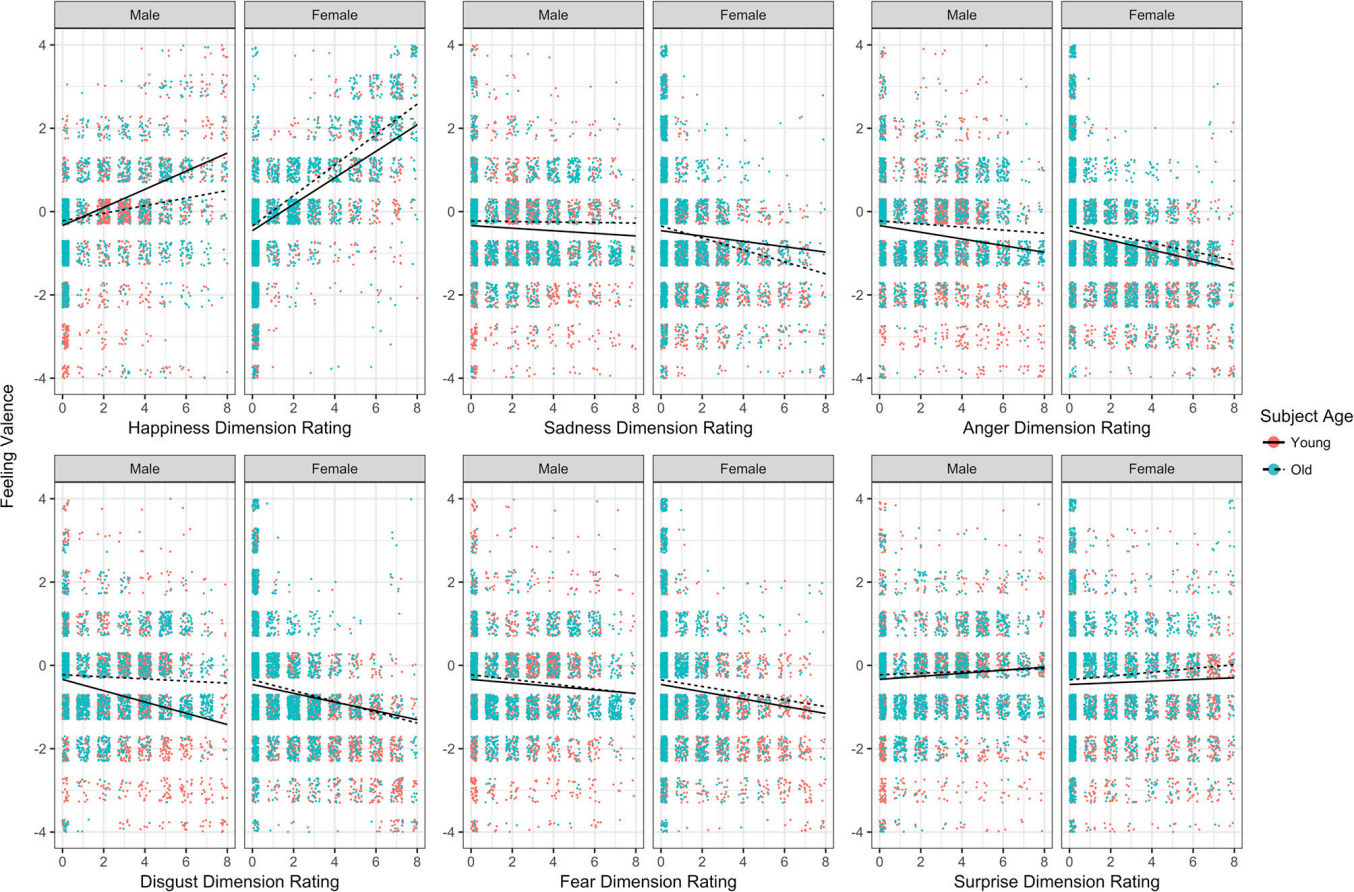
Jittered scatter plots depicting the relationships between emotion rating intensity and subjectively experienced affect, for each dimension of rated emotion, for young and old, male and female groups. Overlaid regression lines are based on the estimated coefficients from the MCMC model (see Supplementary Table 2).

Individual perceived emotional intensities and subjective affective valence induced by each face stimuli for all participants for each emotion dimension are illustrated in Figure 5 and formally tested using a MCMC regression model (Supplementary Table 3). Focusing first on young male responses as the baseline, higher ratings of face happiness and surprise were associated with more positive valences experienced (Supplementary Table 3, rows 2 and 7), as expected. Higher ratings of sadness, anger, disgust, and fear in faces were associated with more negative valences experienced (Supplementary Table 3, rows 3–6). In comparison to young males, older males evinced significantly weaker associations between their ratings of face happiness, anger, and disgust with subjective affect experienced (Supplementary Table 3, rows 10, 12, and 13). In comparison to young males as well, young females evinced significantly stronger associations between their ratings of face happiness, sadness, anger, and fear with subjective affect experienced (Supplementary Table 3, rows 16, 17, 18, and 20). Finally, relative to older males and young females, older females showed stronger associations between their happiness, sadness, disgust, and surprised face ratings with subjective affect (Supplementary Table 3, rows 22, 23, 25, and 27). Thus, subjective affect in females more closely tracked the perceived emotional intensity in faces compared to males. Moreover, sensitivity of affect to perceived emotion expressions declined in older males but was preserved in older females.

### Computer model dissociated assigned face emotion FACS categories in stimuli

**Figure 6:**
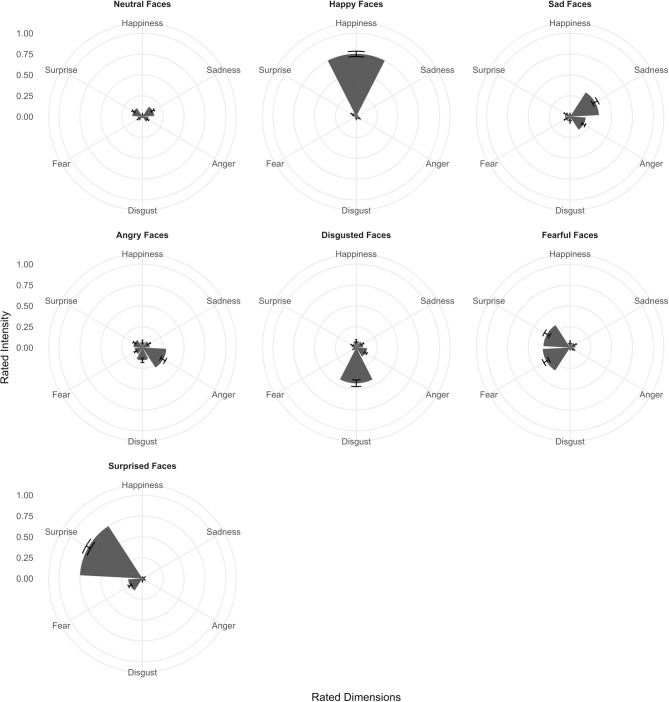
Radar charts showing multidimensional profiles of mean Face Reader ratings across six emotions (happiness, sadness, anger, disgust, fear, and surprise) for happy, sad, angry, disgusted, fearful, surprised, and neutral face expression stimuli categories. Error bars denote standard errors. The maximal intensity of ratings in Face Reader was 1 instead of 8.

**Figure 7:**
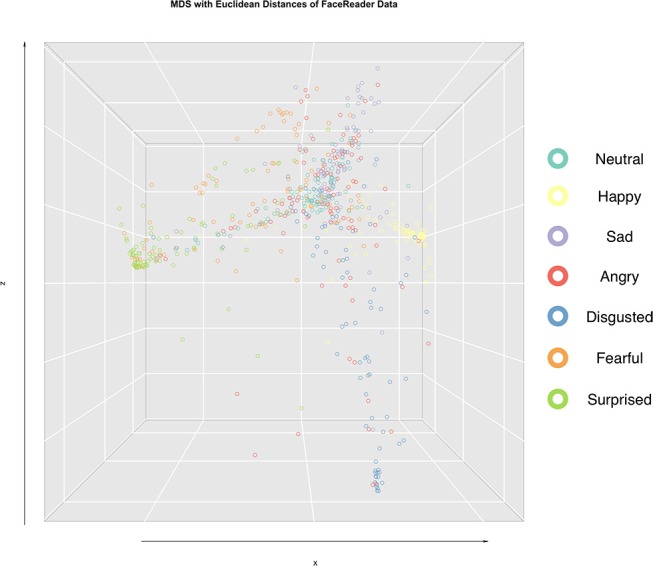
Emotion rating profiles (six emotion dimensions) for each face stimuli from Face Reader were projected in a 3-D space based on multi-dimensional scaling (MDS) analysis. Face stimuli positions in the 3-D space were determined using Euclidean distances (see Methods). Face stimuli position markers were color-coded according to the assigned emotion category from the FACS approach.

**Figure 8:**
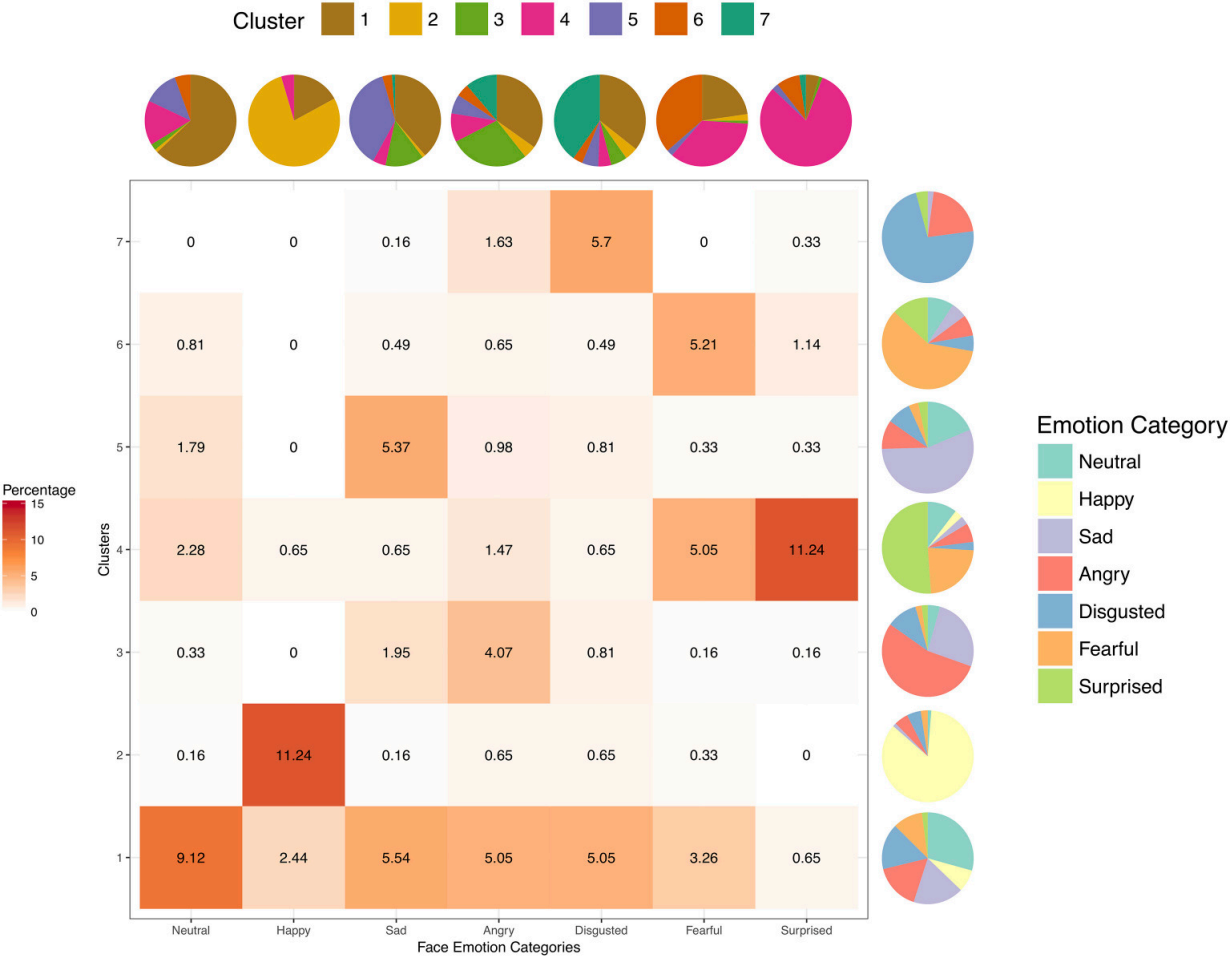
The heat map depicts percentages of all face stimuli in each of the seven face categories for each cluster obtained from hierarchical clustering analysis (cut off at seven clusters) of Face Reader's multi-dimensional ratings. The pie charts represent the marginalized proportions of composition within each cluster (next to each row of the heat map) or face emotion category (above each column of the heat map).

In contrast to human Taiwanese participants, emotion dimension ratings of the same face stimuli performed by Face Reader showed distinct multi-emotional rating profiles (Figure 6). Specifically, happy faces were selectively rated highest on happiness, sad faces on sadness, angry faces on anger, disgusted faces on disgust, and surprised faces on surprise. Of these, we note that the least selective profile was for angry faces. Although fearful faces loaded somewhat similarly on surprise and fear, this pattern was still unique in comparison to the other multi-emotional rating profiles. Visual inspection of the MDS projection of the statistical distances between all face stimuli in 3-dimensional space revealed five clusters (Figure 7 and Supplementary Movie 6). Color-coding the assigned emotion category of each face stimulus revealed separable clusters for happy, sad, disgusted, fearful, and surprised faces. Angry faces appeared evenly distributed with sad and disgusted faces. Percentage membership and marginalized proportions from hierarchical clustering analysis of Face Reader ratings are shown in Figure 8 (see Supplementary Figure 2 for the clustering dendrogram). Consistent with the radar graphs and MDS visualizations, Face Reader yielded distinct multi-dimensional emotion ratings that were generally in concordance with assigned face emotion categories of the stimuli. In Figure 8, we note that cluster 1 was the largest cluster that consisted of several face categories. Nevertheless, cluster 2 was mostly and uniquely represented by happy faces, cluster 3 by angry faces, cluster 4 by fearful and surprised faces, cluster 5 by sad faces, cluster 6 by fearful faces only, and cluster 7 by disgusted faces.

## Discussion

In this study, we established an age- and sex-fair database of facial expressions enacted by Taiwanese young and older, male and female, adults using the FACS approach. Critically, we comprehensively evaluated the perceptions of emotions in these face stimuli in young and older, male and female Taiwanese participants as well as the subjective affective valences induced by the facial expressions. Face stimuli and normative rating data are available upon request. Our findings using these well-controlled face stimuli revealed that Taiwanese participants report perceiving < 6 distinct emotion categories despite objective computerized validation that the visual information was present. Moreover, rater age and sex were significant modulators of emotional ratings such that, compared to younger males, older males perceived less intensities in emotion expressions whereas young females perceived higher intensities. Critically, older females perceived remarkably lower emotional intensities than all three groups. Nevertheless, whereas older male subjective affect was less influenced by emotional intensities perceived, older females maintained emotional sensitivity as with their younger counterparts.

That our Taiwanese participants did not clearly distinguish face emotion expressions according to the six basic emotion categories expressed in natural photographs replicates findings from previous studies comparing Western Caucasians with East Asians using computer generated graphics (Jack et al., 2009, 2012) as well as using other face stimuli databases involving mostly young adult East Asians (Biehl et al., 1997). Specifically, we found that Taiwanese face emotion ratings clustered into three groups that could be categorized as (1) happy, (2) fearful and surprised, and (3) sad, angry, disgusted. Some indication that sadness was least distinctive was also observed in the ratings. These groupings are somewhat consistent with previous findings reporting four groupings with the inclusion of a shame or embarrassment category (Jack et al., 2016). It is likely that we did not detect a fourth category of face expression perception in our study because we only used six basic emotions that did not include embarrassment or shame. Nevertheless, as a novel extension, our study provides strong validation that visual features coding for six basic emotions were present in our naturalistic, rather than simulated, photograph stimuli and detected by commercially developed computer algorithms that were based on the FACS AU method. We suggest that future studies might evaluate neurobiological mechanisms underlying the specific AUs that carry culturally preserved vs. variable emotional signals across people groups. For East Asians and Westerners at least, we note that happy faces are quite consistently categorized. A key distinguishing feature of happy faces is the specific and unique inclusion of AU 12 (lip corner puller), which may be a salient global indicator of this emotion across cultures. The evolution of this facial muscle movement in humans then may be less susceptible to experiential modulation and have greater genetic contributions. Angry and disgusted faces share AUs 10, 17, 25, 26, while sad faces share AU 4 and 15 with angry and disgusted faces, respectively. Besides, surprised and fearful faces share AUs 1, 2, 5, 26, and 27, with sad faces also sharing AU 1 with fearful and surprised faces and AU 4 with fearful faces. We also note that both the FACS approach and the database used to train Face Reader were based primarily on Western samples. Taking these together, we suggest that our findings reflect that while East Asians tend to group these FACS-based face emotions together based on the shared facial muscle movements, Westerners may emphasize the distinguishing AUs and dissociate these face emotions instead. Such an interpretation would be consistent with the engagement of more holistic neural face processing in the fusiform face area in East Asians compared to more analytic face processing neural responses in Westerners (Goh et al., 2010a). The FACS is one of the most widely used method to generate or evaluate face emotion expressions in empirical studies (see Table 1). The merit of the FACS is its definition of facial expression categories using facial muscle movements, which provides a more objective means of evaluating subjective perceptions of face stimuli. Nevertheless, our findings point to the importance for future studies to interrogate the neurobiological and socio-psychological basis for culture-related variability in interpreting emotional signals related to these sets of shared facial muscle movements, based on but perhaps extending beyond the FACS.

Consistent with previous findings on age effects on face emotion recognition, we found that older adults gave lower ratings of emotional intensities expressed in faces relative to young adults. However, whereas previous work reported effects of aging on processing negative face expressions only or at least that the effects are selective across face emotions (Suzuki et al., 2007; Riediger et al., 2011; Suzuki and Akiyama, 2013; Di Domenico et al., 2015; Isaacowitz et al., 2017), we found significant decreases in emotional intensity ratings for all emotions. Thus, our findings are more consistent with studies showing East Asian older adults having less positivity bias in emotional experiences compared to Western older adults, resulting in more equal age effects across all emotions (Grossmann et al., 2014). However, we note that male older adults showed clearly different age-related effects on face emotion perception and experience compared to older females. Thus, it is critical that interpretation of age effects in our Taiwanese sample must be considered jointly with sex differences as well.

On this front, our findings are also consistent with findings on greater sensitivity of females to emotional information compared to males (Rotter and Rotter, 1988; McClure, 2000; Thayer and Johnsen, 2000; Hall and Matsumoto, 2004; Montagne et al., 2005; Williams et al., 2009; Sullivan et al., 2017). Young females provided higher ratings of emotional intensities than young males and the associations between emotional ratings with induced subjective affect were also stronger. Interestingly, older females rated negative affective face emotions lower than older males, but maintained ratings for happy faces. This pattern reflects an age-related positivity bias in face emotion perception in Taiwanese females but not males. Nevertheless, in contrast to older males, older females still maintained strong associations between emotional ratings and subjective affective reactions as with their younger counterparts. Future studies are required to determine whether this specific effect in older Taiwanese female emotion perception reflects culture-specific sex differences in age-related changes in life goals (à* la* socio-emotional selectivity theory; Carstensen et al., 1999; Mather and Carstensen, 2005; Carstensen, 2006), or more biological influences such as from hormonal contributions.

We highlight that age and sex differences in face emotion perception and experience in our sample were likely not due to differential exposure to young or older, male and female faces as might be expected with in-group/out-group biases. Apart from males rating female fearful faces with less surprise, we did not find strong group membership biases in ratings of other-age or other-sex faces. Instead, and consistent with previous literature, we found evidence of less distinctive emotional expressions in older adult faces (Borod et al., 2004; Riediger et al., 2011). We also report novel indication of less fearfulness perceived in female relative to male surprised faces across both male and female participants. Differences in perception of emotion expressions in older adult and female faces may stem from actual age and sex differences in facial muscle movement or skin surface texture and binding to underlying muscles associated with a given AU instruction. It is also possible that social stereotypes might bias the interpretation of the same face emotion expressed by persons of different age or sex groups. The latter is difficult to demonstrate, as it requires comparisons across age and sex groups with different age and sex stereotypes. Nevertheless, further study using objective software model assessments of magnitudes of specific AUs elicited in young and old, male and female, faces during expressions might shed light on the extent to which the associated visual features physically differ across age and sex.

Lower correlations between emotional expression intensity ratings and induced subjective affect in older compared to younger males suggests reduced affective empathy in this group, controlling for group differences in ratings. Indeed, studies have reported poorer empathy or theory-of-mind in older compared to younger adults (Moran et al., 2012; Henry et al., 2013; Chen et al., 2014). However, we note that older females showed similar or even stronger emotional empathy relative to even young females (who already showed stronger emotional empathy than young males). These findings suggest that emotional affective reactions and perception of face expression intensities can vary independently. As such, again, interpretations of age differences in empathy or subjective affect induced by facial expressions should be considered in tandem with the non-trivial contribution of sex differences.

With regard to the representativeness of the ratings of our participants to population behaviors, we highlight that each face stimuli was rated by almost a total of 44 participants under highly controlled laboratory settings, albeit some missing ratings in four participants. Moreover, we note that the 95% confidence intervals for significant coefficients of emotion intensity ratings were generally within +/- 1 for the scales which ranged from 0 to 8 (see Supplementary Tables 2, 3). We suggest that this reflects minimal individual differences in our sample ratings. Also, comprehensive statistical analyses revealed reliable and meaningful significant effects based on the given participant sample size that was quite distinct from the computerized algorithm, which was arguably primarily categorizing stimuli based on visual features. Importantly, in keeping with our aim, the participants consisted of two age groups of raters with approximately equal sample sizes, which afforded data to examine whether the rating patterns in younger Taiwanese would be altered in older Taiwanese (for the reasons described in the introduction). Our data showed that while there were age differences, ratings in the young Taiwanese were generally replicated in older Taiwanese. Finally, we emphasize that participant raters provided emotional judgments that were multi-dimensional. That is, participants did not just identify one single or the most prominent emotion expressed by a given face. Rather, it was explicit to participants to carefully distinguish the extent to which a given face exhibited seven different dimensions of emotions (happiness, sadness, anger, disgust, fear, surprise, neutral), simultaneously. For these reasons, we suggest that our sample provided a reasonably valid range of Taiwanese participant responses and our data reflect real and representative differences in face emotion perceptions, at least for the purposes of our study. We suggest that other broader studies might use the database we now provide to empirically poll a larger and more varied sample as needed to test other specific hypotheses.

Indeed, perhaps the most remarkable finding in this study is that the Taiwanese categorizations of face emotion profiles were consistent across young and older, male and female, adults despite age and sex effects. This suggests that the influences of age and sex differences did not modulate culture-related effects on face emotion perception. Rather, in our study, we observed age and sex effects on face emotion perception that were distinct from other studies based on Western samples. Future work is required to determine the specific cognitive mechanisms behind these culture-related influences on age and sex effects in emotional processing. Facial expressions communicate emotional information that is a fundamental aspect of human social interactions. However, human social interactions always occur in local cultural contexts and this indubitably influences the manner in which emotions are facially expressed, viewed, and interpreted (Blais et al., 2008; Jack et al., 2009; Goh et al., in press). With the trend of increasing inter-cultural interactions across the world, assuming universality of communicative signals, while somewhat present (Darwin, 1873; Ekman and Friesen, 1971), may not be viable and there is a need to understand cultural specificities. Establishment of culture-specific, age- and sex-fair database of face emotion expression stimuli is a critical step to this end.

## Author contributions

Y-ZT, D-WL, and JG: designed research; Y-ZT and D-WL: performed research; Y-ZT and D-WL: analyzed data; Y-ZT, D-WL, AS, and JG: wrote the paper; JG provided resources.

### Conflict of interest statement

The authors declare that the research was conducted in the absence of any commercial or financial relationships that could be construed as a potential conflict of interest.
