# Supplementary material for: East Asian Young and Older Adult Perceptions of Emotional Faces From an Age- and Sex-Fair East Asian Facial Expression Database
Source: Front Psychol. 2018 Nov 29;9:2358. doi: 10.3389/fpsyg.2018.02358 (PMC6281963; doi:10.3389/fpsyg.2018.02358)
Supplement: Supplementary file 10 [file Data_Sheet_1.DOCX]

**SUPPLEMENTARY METHODS**

Equation 1:

$$\left[ \begin{matrix} Happiness & Sadness & Anger & Disgust & Fear & Surprise \end{matrix} \right]=\beta_{1}FaceCategory+\beta_{2}RaterAge+\beta_{3}RaterSex+\beta_{4}FaceCategory\times RaterAge+\beta_{5}FaceCategory\times RaterSex+\beta_{6}RaterAge\times RaterSex+\beta_{7}FaceCategory\times RaterAge\times RaterSex+\beta_{8}FaceCategory\times RaterAge\times FaceAge+\beta_{9}FaceCategory\times RaterSex\times FaceSex+0+\mu_{1}\left( 1 | Face \right)+\mu_{2}\left( 1 | Rater \right)+\varepsilon$$

Equation 2:

$$Feeling=\beta_{1}Happiness+\beta_{2}Sadness+\beta_{3}Anger+\beta_{4}Disgust+\beta_{5}Fear+\beta_{6}Surprise+\beta_{7}RaterAge+\beta_{8}RaterSex+\beta_{9}Happiness\times RaterAge+\beta_{10}Sadness\times RaterAge+\beta_{11}Anger\times RaterAge+\beta_{12}Disgust\times RaterAge+\beta_{13}Fear\times RaterAge+\beta_{14}Surprise\times RaterAge+\beta_{15}Happiness\times RaterSex+\beta_{16}Sadness\times RaterSex+\beta_{17}Anger\times RaterSex+\beta_{18}Disgust\times RaterSex+\beta_{19}Fear\times RaterSex+\beta_{20}Surprise\times RaterSex+\beta_{21}Happiness\times RaterAge\times RaterSex+\beta_{22}Sadness\times RaterAge\times RaterSex+\beta_{23}Anger\times RaterAge\times RaterSex+\beta_{24}Disgust\times RaterAge\times RaterSex+\beta_{25}Fear\times RaterAge\times RaterSex+\beta_{26}Surprise\times RaterAge\times RaterSex+\mu_{1}\left( 1 | Face \right)+\mu_{2}\left( 1 | Rater \right)+\varepsilon$$
