# Supplementary material for: East Asian Young and Older Adult Perceptions of Emotional Faces From an Age- and Sex-Fair East Asian Facial Expression Database
Source: Front Psychol. 2018 Nov 29;9:2358. doi: 10.3389/fpsyg.2018.02358 (PMC6281963; doi:10.3389/fpsyg.2018.02358)
Supplement: Supplementary file 11 [file Data_Sheet_2.PDF]

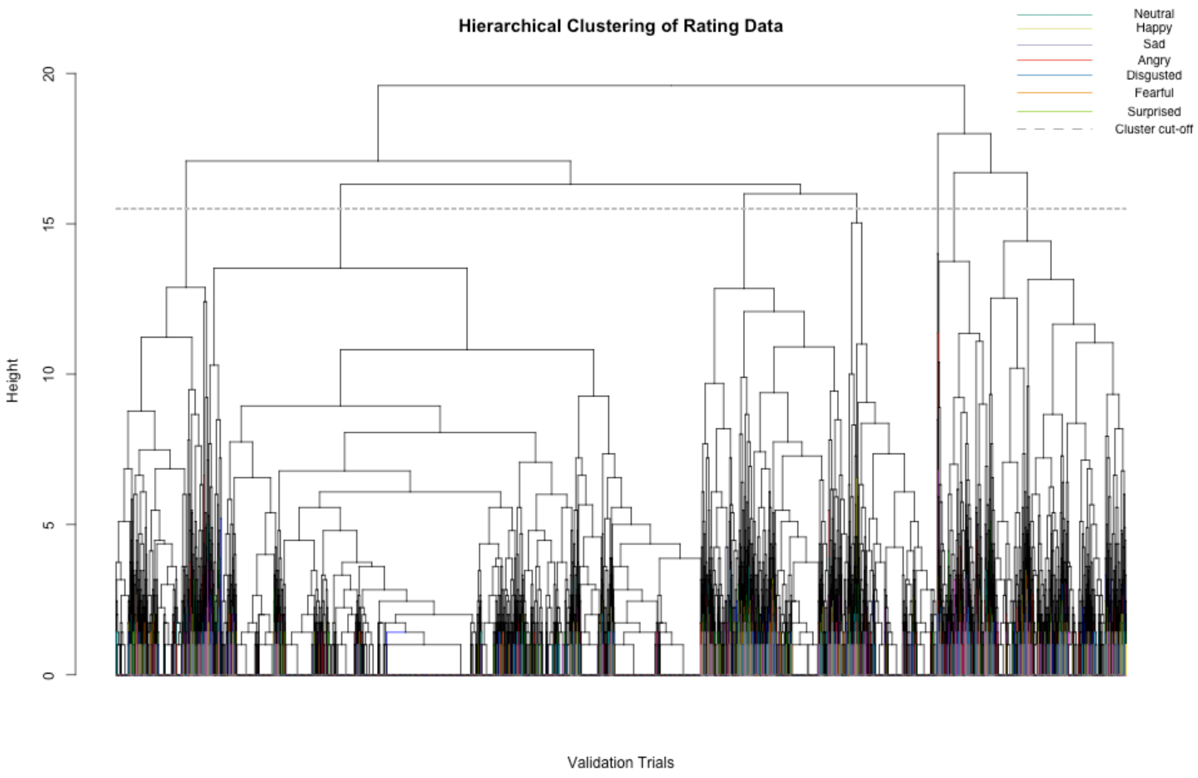

Supplementary Figure 1. Hierarchical clustering result of all ratings trials performed by participants. The leaves of this dendrogram were colored according to original assignments of FACS emotion categories. The dashed line denotes the cut-off height for seven clusters.

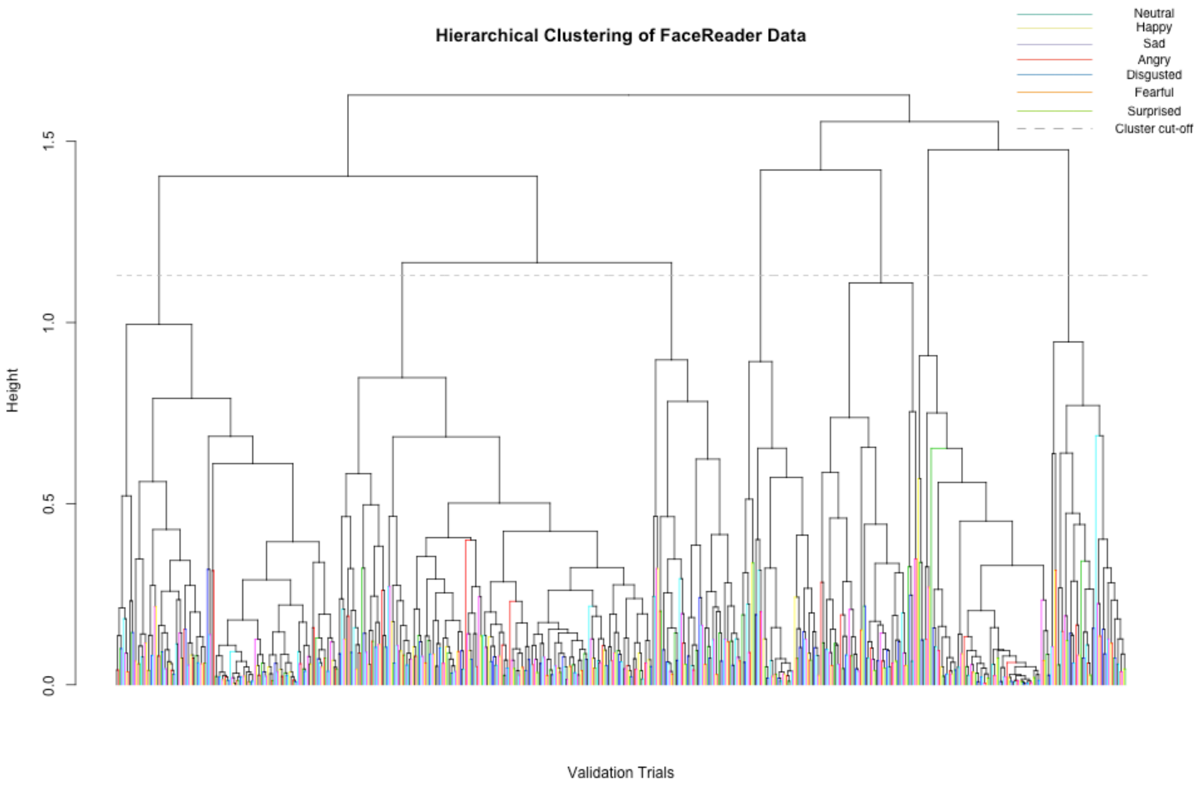

Supplementary Figure 2. Hierarchical clustering result of validation done by Face Reader. The leaves of this dendrogram were colored according to original assignments of FACS emotion categories. The dashed line denotes the cut-off height for seven clusters.
